# *Cephalaria transsylvanica*-Based Flower Strips as Potential Food Source for Bees during Dry Periods in European Mediterranean Basin Countries

**DOI:** 10.1371/journal.pone.0093153

**Published:** 2014-03-27

**Authors:** Giovanni Benelli, Stefano Benvenuti, Nicolas Desneux, Angelo Canale

**Affiliations:** 1 Insect Behaviour Group, Department of Agriculture, Food and Environment, University of Pisa, Pisa, Italy; 2 Agronomy and Agro-ecosystem Management Section, Department of Agriculture, Food and Environment, University of Pisa, Pisa, Italy; 3 French National Institute for Agricultural Research (INRA), Sophia-Antipolis, France; Ghent University, Belgium

## Abstract

The introduction of sown wildflower strips favours the establishment of pollinator communities, with special reference to social Apoidea. Here, we evaluated the late summer flowering *Cephalaria transsylvanica* as suitable species for strips providing food for pollinators in paucity periods. *C. transsylvanica* showed no particular requirements in terms of seed germination and growth during summer. This plant had an excellent potential of self-seeding and competitiveness towards weed competitors. *C. transsylvanica* prevented from entomophilous pollination showed inbreeding depression, with a decrease in seed-set and accumulation of seed energy reserves. However, *C. transsylvanica* did not appear to be vulnerable in terms of pollination biology since it had a wide range of pollinators including bees, hoverflies and Lepidoptera. *C. transsylvanica* was visited mainly by honeybees and bumblebees and these latter pollinators increased their visits on *C. transsylvanica* flowers during early autumn. This plant may be useful as an abundant source of pollen during food paucity periods, such as autumn. We proposed *C. transsylvanica* for incorporation into flower strips to be planted in non-cropped farmlands in intensively managed agricultural areas as well as in proximity of beehives. The latter option may facilitate the honeybees collecting pollen and nectar for the colony, thereby ensuring robustness to overcome the winter season.

## Introduction

The biodiversity and populations of insect pollinators are in substantial decline [1], [2]. Various wild bee species have suffered serious declines [3] and in several cases they have disappeared from their natural habitats [4]. Much attention has been focused on managed honey bees (*Apis mellifera* L.) losses, since their strong population decline is a serious threat to the stability and yield of food crops [5], [6]. A single factor has not been identified to explain the decline of both managed and wild bees and probably multiple factors are likely to be involved. Honey bees have suffered severe losses particularly since 2006–2007 in the USA, when a syndrome called Colony Collapse Disorder (CCD) was firstly described by Oldroyd [7]. The decline of honeybees seems to be due to multiple causes including (*i*) the occurrence of epidemiological factors affecting honeybee health, including disease and parasites [8], [9], (*ii*) the degradation and fragmentation of habitats in intensively managed agricultural landscapes [10], [11], (*iii*) the loss of flower rich plant communities associated with traditional landscape uses [12] and (*iv*) the negative side effects of widespread use of agricultural pesticides [13].

To overcome the pollinators' decline, several tools have been proposed. It has been demonstrated that the communities of flower-visiting insects can be enhanced thanks to field margins, hedges [14], other buffer zones [15] and set-aside fields [16], [17]. Indeed, such areas offer a suitable environment for soil-nesting bee pollinators and Lepidoptera that require particular plant species for oviposition [18]. Moreover, the introduction of flower strips into agricultural landscape may promote the establishment of pollinator communities [19], including butterflies [20] and cavity-nesting Hymenoptera [21], with special reference to honeybees [22] and bumblebees [23]; it may happen also in case of urban ecosystems [24]. Hoverflies are also attracted by some flowering strips, such as alyssum [25]. The use of native wildflowers within or around intensely farmed landscapes helps to sustain pollinator biodiversity, particularly the specialized pollinators linked to specific plants [26]. It could also promote various ecosystem services (see Wratten et al. [2] for a thorough review).

In extensive agricultural areas of European Mediterranean basin countries (e.g. central and southern Italy; southern France and Spain), most wildflower species are micro-thermal and they senesce during late spring. Even if other wildflower blooms are available in the summer, these species hardly grow up in the mentioned extensive agricultural areas, due to little soil fertility and difficult climatic conditions [27]. Overall, there are very few plant species able to grow and bloom during the summer months (notably late in the summer). This could lead to a strong shortage of pollen and nectar resources available to bees. Therefore late summer-flowering wildflowers may have a crucial role for the survival of pollinators during periods characterized by drought and/or food paucity.

**Figure 1:**
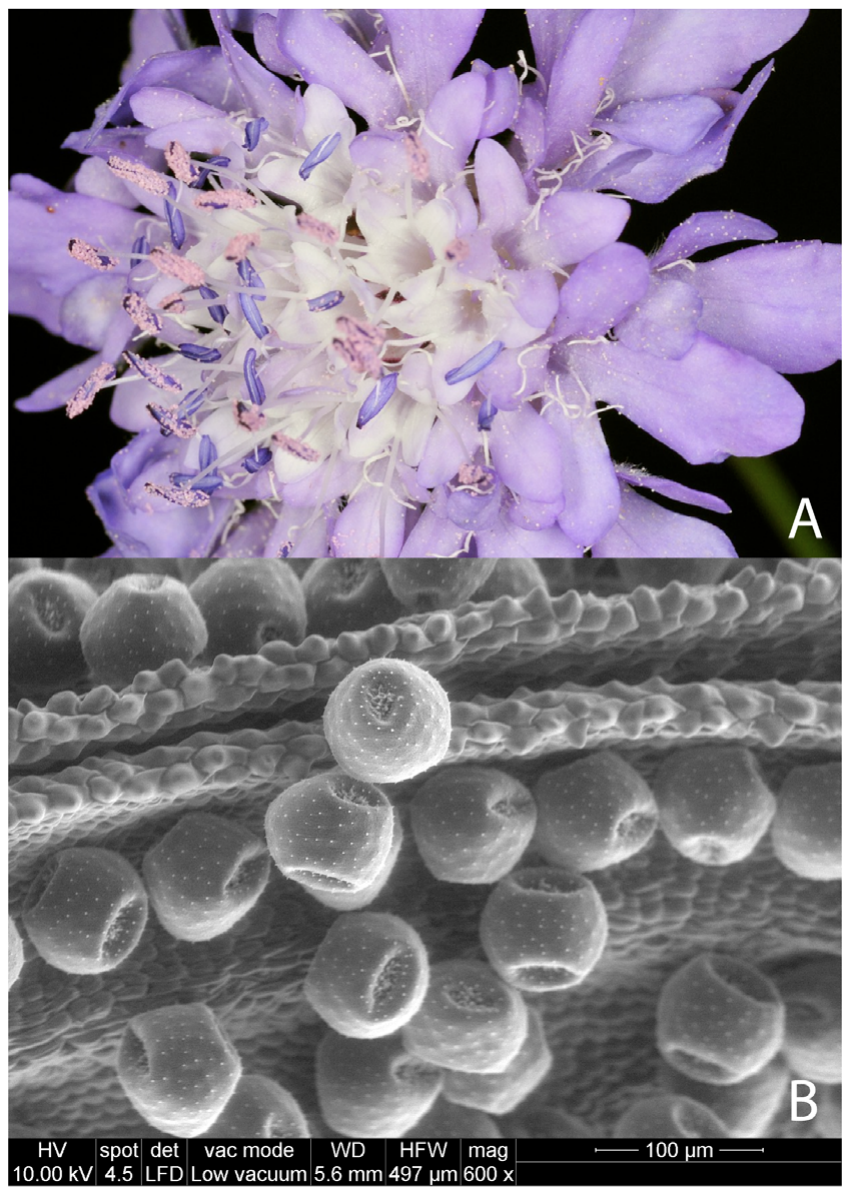
Flowers of *Cephalaria transsylvanica* (A) and scanning electron micrograph of the pollen (B).

Among Dipsacaceae plants, the genus *Cephalaria* Schrad. ex Roem. & Schult includes 93 species of herbaceous plants, native to southern Europe, East Asia, and North and Central Africa [28]. *Cephalaria transsylvanica* (L.) Schrader (Dipsacaceae) is an annual, late-summer flowering species with lilac flowers and pink-coloured pollen [29] (**Figure 1**). It commonly grows in European Mediterranean basin countries (e.g. Turkey, Greece, southern of Italy, France and Spain), as well as in Romania and some parts of Russia. *C. transsylvanica* is able to develop in areas characterized by poor soil fertility and summer drought. It has been used in medicine owing to its wide range of biological activities, including hypothermic, alleviative, relaxant and anti-infective activities [30]. *C. transsylvanica* flowers have been preliminarily reported as pollen sources for several insects, including honeybees [31] and bumblebees [32]. In European Mediterranean basin countries, *C. transsylvanica* usually blooms during late summer and autumn (i.e. from June to early November).

Among the other plants belonging to the Dipsacaceae family, *Dipsacus fullonum* Linnaeus is currently the main species used for flowering strips, due to the abundant nectar production [33], [34], [35]. However, in European Mediterranean basin countries, it blooms mainly during mid-summer. To the best of our knowledge, no late blooming plant species have been proposed to improve the composition of flower strips. We hypothesize that the late flowering *C. transsylvanica* may be a key source of pollen to many flower-visiting insects. Its presence in flowering strips in non-crop farmlands could help pollinators, notably those belonging to the Apoidea family, to overcome periods of low food availability (e.g. early autumn) when flowering plants are scarce and bees need to accumulate protein-rich food before the winter. On this basis, the present study is aimed to determine if *C. transsylvanica* would be suitable as a rustic species for improving the composition of flowering strips in non-cropped farmlands used in European Mediterranean basin countries. We also investigated its ability to attract insect pollinators through the production of pollen in late summer and autumn.

## Materials and Methods

### General observations and germoplasm collection

All experiments were carried out in the experimental fields of the University of Pisa (Italy). No specific permissions were required for these activities. The study did not involve endangered/protected species. Seeds of *C. transsylvanica* were collected during Autumn 2010 in field margins of the University of Pisa farmlands (43°70′N 10°43′E; 5 m) used for cultivation of winter cereals. Climatic condition of the experimental site was provided in **Figure S1**. The soil was poor in nutrients and organic matter, with sandy-loam texture and dry during the summer. Seeds were collected from fully senescent flowers and stored at the University of Pisa laboratories (18°C and 60% R.H., natural photoperiod) until their use. The weight of the *C. transsylvanica* seeds was determined according to the ISTA method [36].

### Cultivation of *Cephalaria transsylvanica* strips

*C. transsylvanica* was cultivated in the experimental fields of the Department of Agriculture, Food and Environment of the University of Pisa (43°70′N, 10°43′E) in a sandy-soil (sand 78%; lime 14%; clay 8%; pH 8.5; organic matter 1.2%). A ground strip (3×21 m) of an uncultivated area has been demarcated and three harrowing were made, during summer and autumn 2011, in order to reduce the pre-existing weed seed bank. In November 15th 2011 seeds of *C. transsylvanica* were hand-sown (3 g per square meter) and a rolling treatment followed, allowing the seed-soil contact and enhancing the seed germination process [37]. The parcel was divided into three sub-plots (3×7 m) to perform biometric measures adopting a randomized block experimental design.

The analysis of germination of *C. transsylvanica* seeds in laboratory conditions was carried out in Petri dishes (12∶12 (L∶D photoperiod), alternating temperatures of 15–25°C (L∶D, respectively)). The field evaluation of the percentage of emergence has been carried delimiting some small areas (10 sub-plots of 20×20 cm). Every three days the number of emerged seedlings (i.e. the appearance of the cotyledons) was noted, until the emergence dynamics stood on constant values (about 1 month after sowing). The rate of emergence (i.e. number of emerged seedlings/number of distributed seeds) was calculated. From May to November, the number of inflorescences in *C. transsylvanica* strips was quantified three times per month. A metal frame (30×30 cm) was placed over the plants allowing a non-destructive counting of the number of inflorescences. The height of inflorescences was also measured. At the end of both years of cultivation (i.e. 2012 and 2013), we quantified the number of surviving plants, through field observations conducted in the first week of October. Since *C. transsylvanica* is a very rustic species, it was not necessary to provide fertilization or irrigation treatments. In November 2012, the aerial plant parts were cut at 5 cm from the soil, since they were fully senescent. This was done to provide space for germination and emergence of seeds fallen to the ground after plant senescence. To quantify the biomass of *C. transsylvanica*, aerial plant parts were placed in a ventilated stove (60°C) for a week, until complete drying, then weighted.

### Role of entomophily on *Cephalaria transsylvanica* seed-set

To establish the requirement for insect pollination for seed-set, some *C. transsylvanica* inflorescences were made inaccessible to visiting insects during August 2012 and 2013. Following the methods described by Jacobs et al. [38], the buds of some inflorescences were “bagged” (BG) in pre-flowering with tulle mesh bags. Tulle is sufficiently fine to prevent insects from reaching flowers, but has a coarser weave (1.2 mm) over nylon or muslin (0.5–0.7 mm), allowing more airborne pollen to pass through, whilst still being insect-proof. Others *C. transsylvanica* inflorescences, the “open pollination” (OP) ones, were left open to flower-visiting insects. After senescence, 20 BG inflorescences were harvested from each of the three sub-plots (total: 60 BG inflorescences/year) and compared with 20 OP inflorescences per subplot (total: 60 OP inflorescences/year). The plant material from both treatments was collected and transferred to the University of Pisa laboratories. For each inflorescence, the number of seeds and their relative weight were noted.

### Insects foraging on *Cephalaria transsylvanica* strips

Investigations were carried out during *C. transsylvanica* flowering (August and September 2013). Insects were directly observed during foraging activity on *C. transsylvanica* flowers, then captured using an entomological net. From August 15th to September 30th, twelve samples were carried out (two samplings/week). For each sample date, two observation periods were chosen: morning (from 10:00 to 12:00) and early afternoon (from 14:00 to 16:00). Collected specimens were kept separately in plastic test tubes then dry mounted and identified at a specific level.

Four specimens for each species were observed with an environmental scanning electron microscope (ESEM, hereafter) (FEI Quanta 200, Hillsboro, USA) to ensure the presence of *C. transsylvanica* pollen on the insect's body [39], legitimating each insect species as pollinator for *C. transsylvanica* [40], [41]. Voucher specimens of all species were stored in entomological boxes and kept at the Entomological Section of the University of Pisa.

### Data analysis

Biomass production data were analyzed using a General Linear Model (GLM) with two factors (JMP 7, SAS, 1999): y_j_ = μ+P_j_+Y_j_+P*Y_j_+e_j_ in which y_j_ is the observation, μ is the overall mean, P_j_ the plant species (i.e. *C. transsylvanica*, weed competitors; j = 1–2), Y_j_ the year of cultivation (i.e. 2012, 2013; j = 1–2), P_j_*Y_j_ the interaction between the plant species and the year of cultivation, and e_j_ the residual error. Means were compared by Tukey-Kramer HSD post-hoc test (at the *P*<0.05 significance level).

Data on the role of entomophily on *C. transsylvanica* seed set (i.e. seed number and weight in BG and OP inflorescences) were processed using the above-described GLM with two factors, the pollination (i.e. BG, OP; j = 1–2), the year of cultivation (i.e. 2012, 2013), and their interaction. Averages were separated by Tukey-Kramer HSD test. Data on flowering dynamics (i.e. number of inflorescences per square meter) were analysed by ANOVA (CoHort software, Minneapolis, USA) followed by the Student–Newman–Keuls test (at the *P* = 0.05 as level of significance) for separation of means.

Data on the abundance of the three major flower-visiting insects over time [*A. mellifera*, *Bombus pascuorum* (Scopoli) (Hymenoptera: Apidae) and *Halictus scabiosae* (Rossi) (Hymenoptera: Halictidae)] were analyzed using a weighted generalized linear model with two fixed factors (JMP 7, SAS, 1999): y = Xß+ε where y is the vector of the observations (i.e. abundance of each insect species), X is the incidence matrix, ß is the vector of fixed effects (i.e. the insect species, the time of capture) and ε is the vector of the random residual effects.

## Results

### Cultivation of *Cephalaria transsylvanica* strips

**Figure 2:**
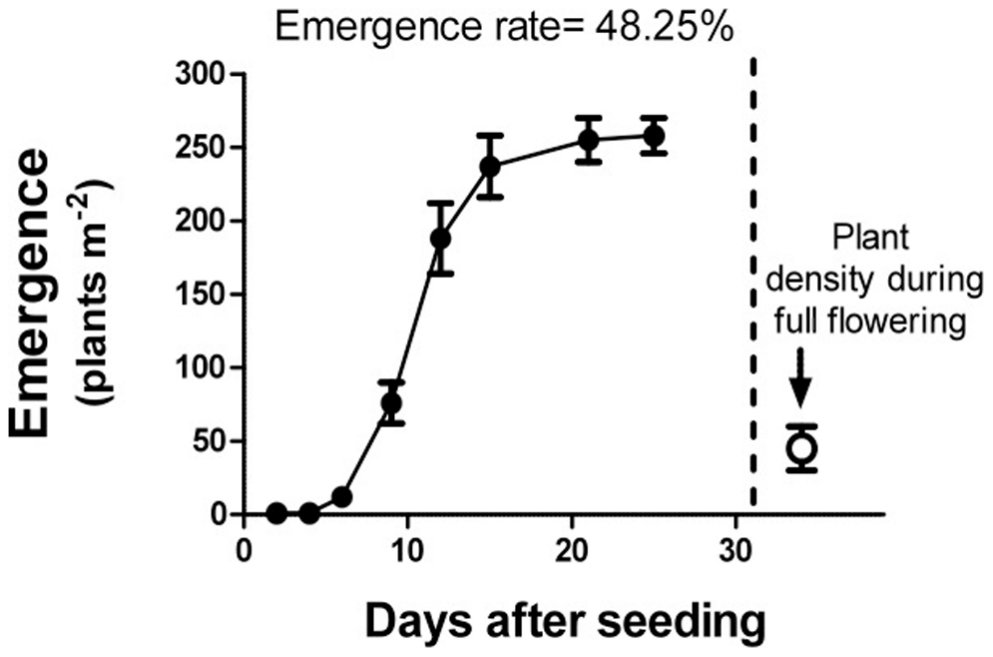
*Cephalaria transsylvanica* cultivation: seed survival emergence and density of plants over time. Seedling date: November 15^th^ 2011. T-bars indicate the standard errors.

The weight of 1000 seeds of *C. transsylvanica* was 5.2±0.3 g. **Figure 2** showed the dynamics of emergence of *C. transsylvanica* seeds during the autumn 2011. After 10 days from sowing, the quantity of emerged seedlings was higher than 70 plants per square meter. It took about 3 weeks to reach about 250 seedlings per square meter. The final number of plants was about 50 plants pr square meter. The seedling survival was 48.25±2.60% (**Figure 2**), while germination of seeds in vitro reached 62.50±3.40%.

**Figure 3:**
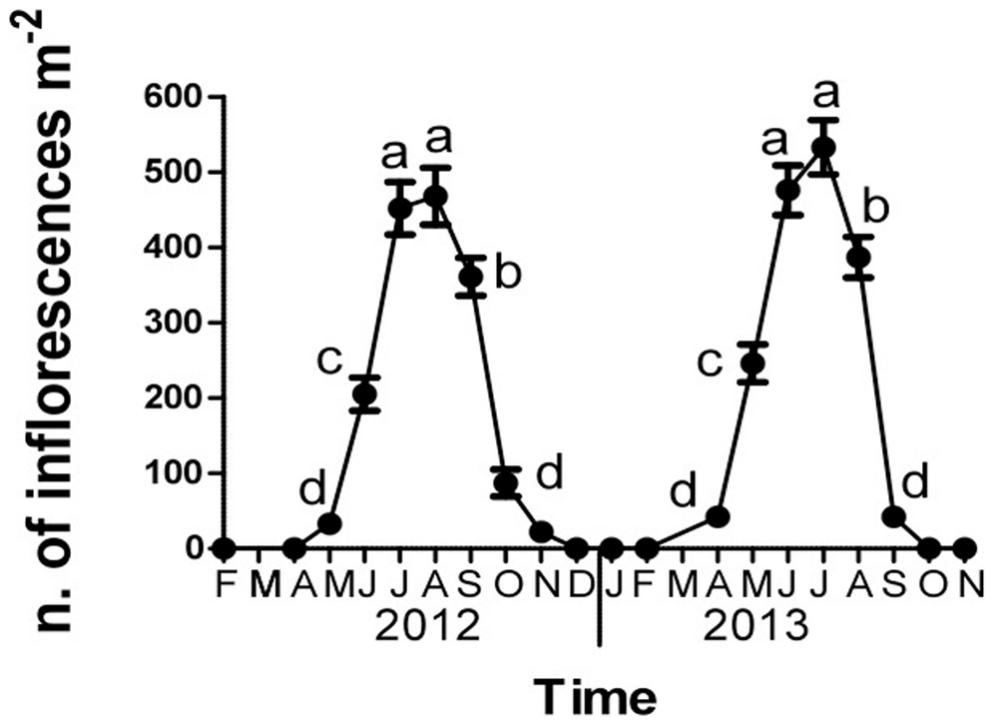
*Cephalaria transsylvanica* cultivation: flowering dynamics during 2012 and 2013. T-bars bars indicate standard errors. Different letters indicate significant differences among the number of inflorescences (ANOVA, Student–Newman–Keuls test, P<0.01).

In both years of cultivation, the maximum of *C. transsylvanica* plants flowering occurred during July and August, with about 500 inflorescences per square meter. It was significantly higher than number of inflorescences recorded in previous and following months (**Figure 3**). The number of inflorescences was still relatively high during June (200–250 inflorescences per square meter) and September (400 inflorescences per square meter) (**Figure S2**). By contrast, in May and October it was lower than 100 inflorescences per square meter. In both years, the inflorescence exceeded 1 m height; it reached 1.5 m during the second year of cultivation (**Table S1**), and the inflorescences are usually arranged in apical positions.

Concerning the total biomass production by *C. trannsylvanica* and competitor weeds, a significant effect of the plant species (*F* = 210.238; *d.f.* = 1; *P*<0.001) and of the interaction plant species*year (*F* = 5.931; *d.f.* = 1; *P* = 0.021), but not of the year (*F* = 1.256; *d.f.* = 1; *P* = 0271), was detected. Both *C. transsylvanica* (687 g per square meter in 2013 *versus* 548 g per square meter in 2012; n.s.) and weeds (23 g per square meter in 2013 *versus* 75 g per square meter in 2012; n.s.) produced similar quantities of biomass in the two cultivation years. In both years, weed competitors were represented by *Digitaria sanguinalis* (L.) Scop., *Helminthotheca echioides* (L.) Holub, *Polygonum aviculare* Linnaeus, *Setaria viridis* (L.) P. Beauv. and *Sonchus oleraceus* Linnaeus.

### Role of entomophily *on Cephalaria transsylvanica* seed-set

**Table 1** showed the seed-set occurring in BG and OP inflorescences. Absence of entomophily caused a decrease of about 30% in the number of seeds (*F* = 290.445; *d.f.* = 1; *P*<0.001), independently from the year of cultivation (*F* = 1.676; *d.f.* = 1; *P* = 0.197). Also the effect of the interaction pollination*year was significant (*F* = 6.353; *d.f.* = 1; *P* = 0.012). The unit weight of the seeds was significantly less (about 20%) under BG conditions (**Table 1**) (*F* = 134.516; *d.f.* = 1; *P*<0.001). This parameter is also affected by the year of cultivation (*F* = 7.419; *d.f.* = 1; *P* = 0.007), but not by the interaction pollination*year (*F* = 1.747; *d.f.* = 1; *P* = 0.188).

**Table 1:** Reproductive performances of “open-pollination” (OP) and “bagged” (BG) inflorescences of *Cephalaria transsylvanica* in terms of seed production and relative seed weight.

Year	Seeds per inflorescence (n)		1.000 seed weight (g)	
	OP	BG	OP	BG
2012	35.7 b	25.2 c	5.1 a	4.1 c
2013	38.5 a	24.4 c	5.3 a	4.4 b

Values followed by different letters are significantly different (General Linear Model, Tukey HSD test, *P*<0.05).

### Insects foraging on *Cephalaria transsylvanica* strips

**Figure 4:**
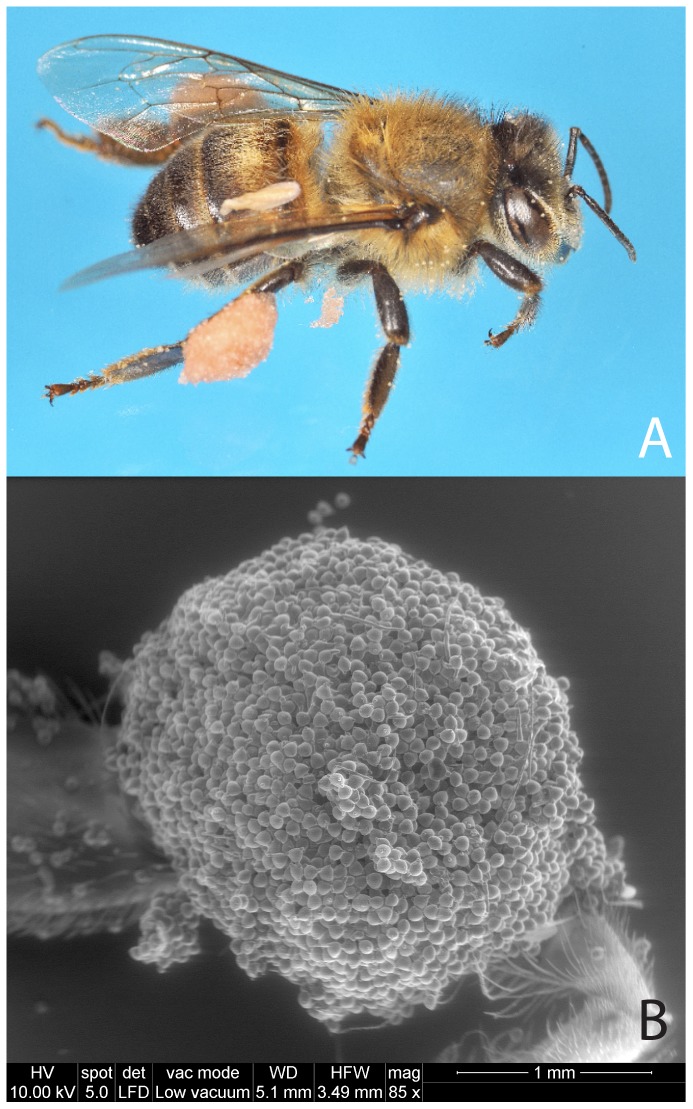
A honeybee, *Apis mellifera*, after foraging on *Cephalaria transsylvanica* flowers (A). The pink-coloured pollen grains of *C. transsylvanica* have been mass-packed in the pollen baskets located on the third pair of legs (red arrow). Scanning electron micrograph (external view) of a *Cephalaria transsylvanica* pollen mass packed in the pollen basket located on a leg of *A. mellifera* (**B**).

*C. transsylvanica* flowers were visited for pollen and nectar by insect species belonging to Hymenoptera, Diptera and Lepidoptera (**Table 2**). Among bees, generalist social species, mainly *A. mellifera* (**Figure 4**) and *B. pascuorum* (**Figure S3**), dominated. The presence of honeybees, bumblebees and sweatbees increased from late summer to early autumn (*X^2^* = 35.158; *d.f.* = 2; *P*<0.001), regardless from the pollinator species (*X^2^* = 0.235; *d.f.* = 2; *P* = 0.889) and from the interaction pollinator species*time period (*X^2^* = 12.090; *d.f.* = 4; *P* = 0.877). Concerning Diptera, five species of Syrphidae were recorded as foragers on *C. transsylvanica* flowers (**Figure S4**). In addition, various Lepidoptera species (**Table 2**) were also recorded on *C. transsylvanica* flowers, notably individuals belonging to the Papilionidae [e.g. *Iphiclides podalirius* (Linnaeus)] and the Pieridae [e.g. *Pieris brassicae* (Linnaeus)] families.

**Table 2:** Insects foraging on *Cephalaria transssylvanica* strips cultivated in the experimental fields of the University of Pisa, Italy (43°70′N 10°43′E; 5 m) during late summer and early autumn.

Order, family and species	August 15^th^–30^th^, 2013	September 1^st^–15^th^, 2013	September 15^th^–30^th^, 2013	N
Hymenoptera, Apidae				
*Apis mellifera* Linnaeus, 1758	6	8	19	33
*Bombus pascuorum* (Scopoli, 1763)	5	10	27	42
*Bombus sylvarum* (Linnaeus, 1761)	2	5	7	14
*Xylocopa violacea* Linnaeus, 1758	1	1	2	4
Hymenoptera, Halictidae				
*Halictus scabiosae* (Rossi, 1790)	7	7	21	35
Hymenoptera, Megachilidae				
*Megachile flabellipes* Pérez 1895	0	1	2	3
Diptera, Syrphidae				
*Eristalis anthophorina* (Fallén, 1817)	0	0	2	2
*Eristalis tenax* (Linnaeus, 1758)	2	2	2	6
*Episyrphus balteatus* De Geer, 1776	2	2	4	8
*Syrphus ribesii* (Linnaeus, 1758)	0	0	1	1
*Volucella zonaria* (Poda, 1761)	2	3	5	10
Lepidoptera, Hesperiidae				
*Ochlodes sylvanus* (Esper, 1777)	0	1	3	4
Lepidoptera, Lycaenidae				
*Polyommatus icarus* (Rottemburg, 1775)	0	2	1	3
Lepidoptera, Nymphalidae				
*Maniola jurtina* (Linnaeus 1758)	5	3	2	10
Lepidoptera, Papilionidae				
*Iphiclides podalirius* (Linnaeus, 1758)	2	2	4	8
Lepidoptera, Pieridae				
*Pieris brassicae* (Linnaeus, 1758)	2	3	5	10
**Total identified**	**36**	**50**	**107**	**193**

For each period, the abundance of species is reported. N = total number of observed insects.

## Discussion

Our results showed that *C. transsylvanica* is a rustic species, with no peculiar requirements in terms of growth during dry summer periods characterizing European Mediterranean basin countries. This plant has an excellent potential of self-seeding and competitiveness towards weed competitors and does not appear to be particularly vulnerable in terms of pollination biology, since it is served by a wide range of insect pollinators. Interestingly, some Apoidea pollinators increase their visits for pollen on *C. transsylvanica* flowers during early autumn, highlighting the potential value of this flowering for bees during food paucity periods.

Agronomic results highlighted that, even if the number of emerged seedlings reached about 250 seedlings per square meter, the final number of plants reached only 50 plants per square meter. This could result from intra-specific competition among plants as well as to allelopathic inhibition caused by the release of toxic substances (e.g. as reported in alfalfa) [42]. On the other hand, the high number of seeds that we used during our experiments was a conservative choice to avoid an excessive thinning of young plants due to biotic (e.g. phytophagous pests and animal trampling) and abiotic stress (e.g. cold, drought, water shortage) [43]. Even if the abundant *C. transsylvanica* seed rain originated about the double of the seedlings emerged in the previous year, the final density was about 50 plants per square meter, in both cultivation years. Many seeds that we tested did not germinate, probably because wild species are frequently characterized by high seed dormancy. Furthermore, the difference among *in vivo* (48.25±2.60%) and *in vitro* (62.50±3.40%) emergence rate could be due to many different causes, including the occurrence of both seed dormancy and germination inhibition (via hypoxia) in the soil [44]. The maximum *C. transsylvanica* flowering occurred during July and August. However, the number of inflorescences was abundant also in September. The availability of *C. transsylvanica* pollen in early autumn could be crucial for the survival of pollinators; only few plant species actually provide both food sources to pollinators in the European Mediterranean basin countries [45].

In *C. transsylvanica*, entomophily caused an increase in number and weight of produced seeds. Similarly, a reduction in weight of self-pollinated seeds has been observed in *Scabiosa columbaria* Linnaeus (Dipsacaceae) [46]. This highlights a possible co-evolution to improve gene flow through services of a wide range of pollinators [47]. Particularly, the production of seeds with a reduced amount of endosperm implies less vigour of the offspring and a lower degree of competitiveness of its seedlings in the surrounding plant communities. This latter point has some practical implications in a species such as *C. transsylvanica*, since this plant is a very rustic and we hypothesize that it can be planted and let reproduce year after year by itself in flowering strips. On this basis, a good pollination service by flower-visiting insects may help *C. transsylvanica* individuals to successfully reproduce over years. A shortage of pollinators for prolonged periods can make *C. transsylvanica* reproduction vulnerable, as observed for other species, including *Knautia arvensis* (L.) Coulter [48], [49]. Interestingly, both *C. transsylvanica* and *K. arvensis* are protected against self-pollination within flower heads through protandry, and the likely mechanism for selfing is via geitonogamy among flower heads [49]. On the other hand, other rustic *Cephalaria* species are generally seen as weeds in Mediterranean areas. For instance, Zohary [50] reported that *Cephalaria syriaca* Scrad. ex Roem. & Schult. can become more abundant than its hosting cereal crop. Furthermore, since the seeds of *Cephalaria* spp. show the same size and weight they cannot be sorted out easily from barley grain and this can enhance reseeding. Further research is needed to evaluate if *C. transsylvanica* can invade neighbouring and/or following crops, thus becoming a serious weed for cereal crops.

Concerning insect pollinators, *C. transsylvanica* flowers were visited for pollen by many species, with a dominance of generalist social Hymenopteran species, notably *A. mellifera* and *B. pascuorum*. In agreement with our findings, wild *C. transsylvanica* plants have been preliminarily reported as a food sources for honeybees and bumblebees [31], [32], even if details on the identity of these pollinators and their functional ecology are lacking. Also Benedek [51] observed some bee species foraging on wild *C. transsylvanica* specimens, including *Halictus malachurus* Kirby, *H. calceatus* Scopoli, *H. maculatus* Smith (Hymenoptera: Halictidae) and *Bombus sylvarum* (Linnaeus) (Hymenoptera: Apidae). Apparently, *C. transsylvanica* flowers can be pollinated by most of the long-tongued Apoidea we observed, including rare species, such as *B. pascuorum*. Interestingly, the presence of honeybees, bumblebees and sweatbees increased from late summer to early autumn, pointing out that the foraging of bees on strips of this plant became crucial in food paucity periods, when other blooms are lacking. In this context, the visiting insects probably gain from searching lipid-rich rewards, such as the *C. transsylvanica* pollen [52]. Hoverflies were also recorded as foragers on *C. transsylvanica* flowers and we suppose that their role as pollinators has been probably under estimated in the past [53]. Indeed, adults of Syrphinae and Eristalinae visit of a wide range of flowers and feed most on nectar, using their long proboscis [54]. However, it has been demonstrated that hoverflies can use labellar food furrows to feed on pollen [39], [55], thus improving their diet with a protein-rich food. We recorded various Lepidoptera (e.g. Papilionidae and Pieridae) on *C. transsylvanica* flowers. These insects have a long proboscis that enables them to visit flowers with nectaries hidden in an elongated calyx, such as *C. transsylvanica* and other Dipsaceae (e.g. *K. arvensis* and *D. fullonum*). On the other hand, the pollen transport by butterflies seems to be less efficient than Hymenoptera [56] and pollen grains of *C. transsylvanica* have been found on the Lepidoptera mouthparts only occasionally (Benelli G. pers. observ.). Overall, *C. transsylvanica* flowers showed both an ecological and functional generalization, since they can be visited by a wide variety of insects that service plants at a high taxonomic level [57], [58].

Based on our results, it may be possible to use *C. transsylvanica* for providing food sources to bees in flowering strips during dry summer periods and early autumn. That plant species showed no particular requirements in terms of seed germination, growth and water availability during the warmest summer months. It also had an good potential of self-seeding and competitiveness towards weed competitors. *C. transsylvanica* prevented from entomophilous pollination showed inbreeding depression with a decrease in seed-set and accumulation of energy reserves in the seeds. However, this species did not appear to be vulnerable in terms of pollination biology since it had a wide range of pollinators including solitary and social bees, hoverflies and Lepidoptera species. The fact that *C. transsylvanica* was visited largely by honeybees and bumblebees, associated to the increase of these visits during early autumn, may hint that this plant could be useful as an abundant source of pollen during food paucity periods, such as early autumn. On this basis, we propose this species for inclusion in flower strips used in European Mediterranean basin countries. These can be sown both in intensively managed agricultural areas, in order to increase the pollinators' diversity, as well as in close proximity of beehives. The latter use may facilitate the honeybees collecting pollen for the colony, thereby ensuring robustness to overcome the winter season.

## Supporting Information

Figure S1 **Climatic trend (maximum and minimum temperatures, rainfalls) that occurred during the cultivation of** ***Cephalaria transsylvanica*** **at University of Pisa (Italy).**
(TIF)

Figure S2 ***Cephalaria transsylvanica*** **in a cultivated strip at the University of Pisa (Italy), during full flowering (September 2013).**
(TIFF)

Figure S3 **The common carder bee,** ***Bombus pascuorum*** **: scanning electron micrograph of the tarsal region carrying** ***Cephalaria transsylvanica*** **pollen.**
(TIFF)

Figure S4 ***Cephalaria transsylvanica*** **is an abundant source of pollen for different insect species, including hoverflies (Diptera, Syrphidae): (A)** ***Eristalis tenax*** **and (B)** ***Volucella zonaria*** **.**
(TIF)

Table S1 **Height of the** ***Cephalaria transsylvanica*** **inflorescences during the summer of the two cultivation years (2012–2013).**
(DOC)
