# Supplementary figures and images for: Cephalaria transsylvanica-Based Flower Strips as Potential Food Source for Bees during Dry Periods in European Mediterranean Basin Countries
Source: PLoS One. 2014 Mar 27;9(3):e93153. doi: 10.1371/journal.pone.0093153 (PMC3968061; doi:10.1371/journal.pone.0093153)

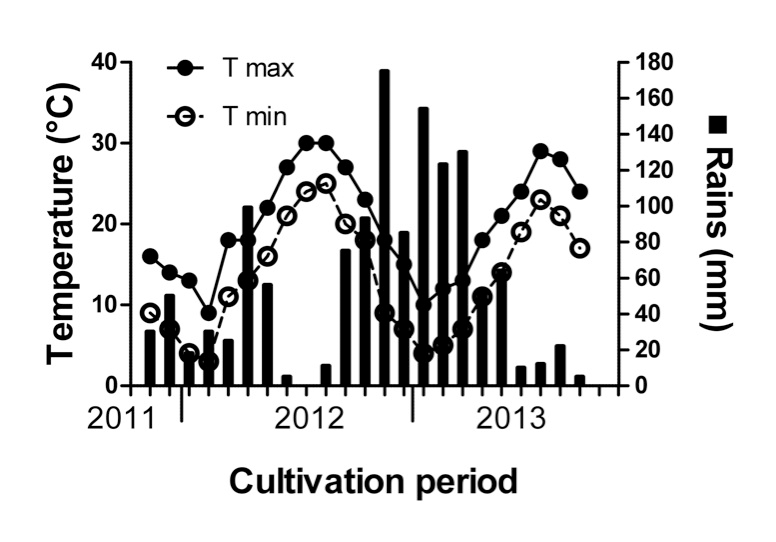

Supplement: Figure S1 — Climatic trend (maximum and minimum temperatures, rainfalls) that occurred during the cultivation of Cephalaria transsylvanica at University of Pisa (Italy). (TIF) [file pone.0093153.s001.tif]

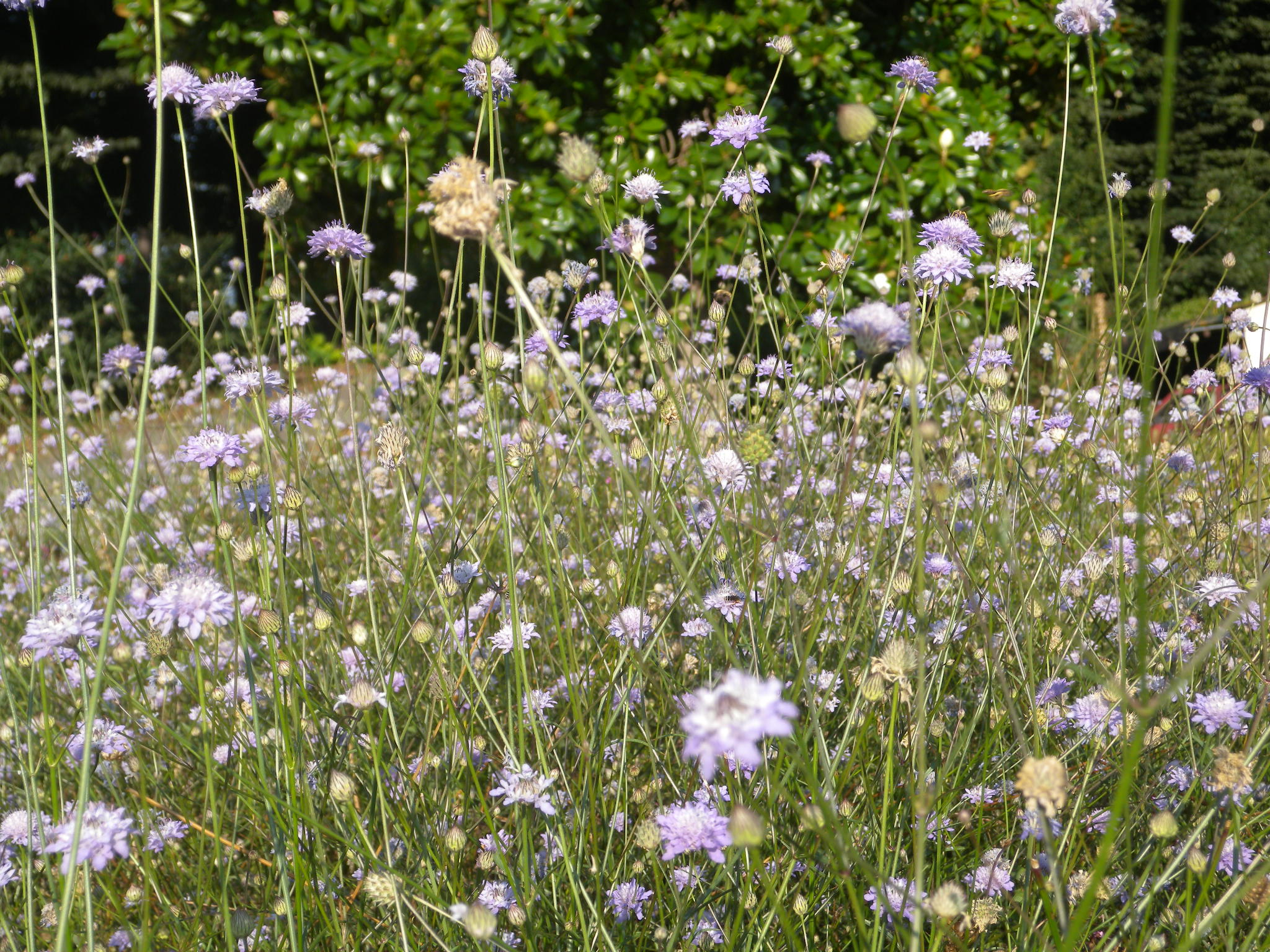

Supplement: Figure S2 — Cephalaria transsylvanica in a cultivated strip at the University of Pisa (Italy), during full flowering (September 2013). (TIFF) [file pone.0093153.s002.tiff]

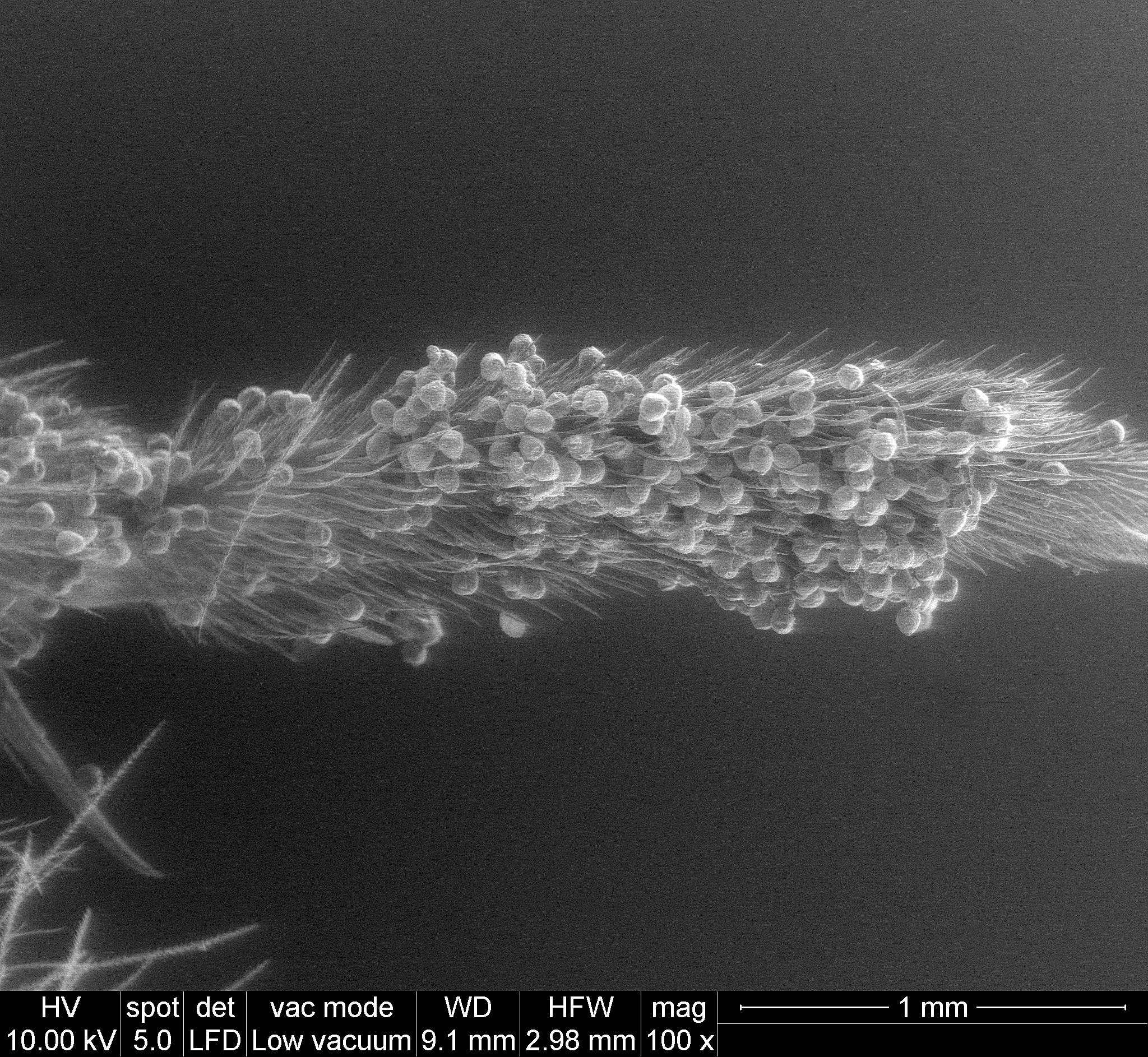

Supplement: Figure S3 — The common carder bee, Bombus pascuorum : scanning electron micrograph of the tarsal region carrying Cephalaria transsylvanica pollen. (TIFF) [file pone.0093153.s003.tiff]

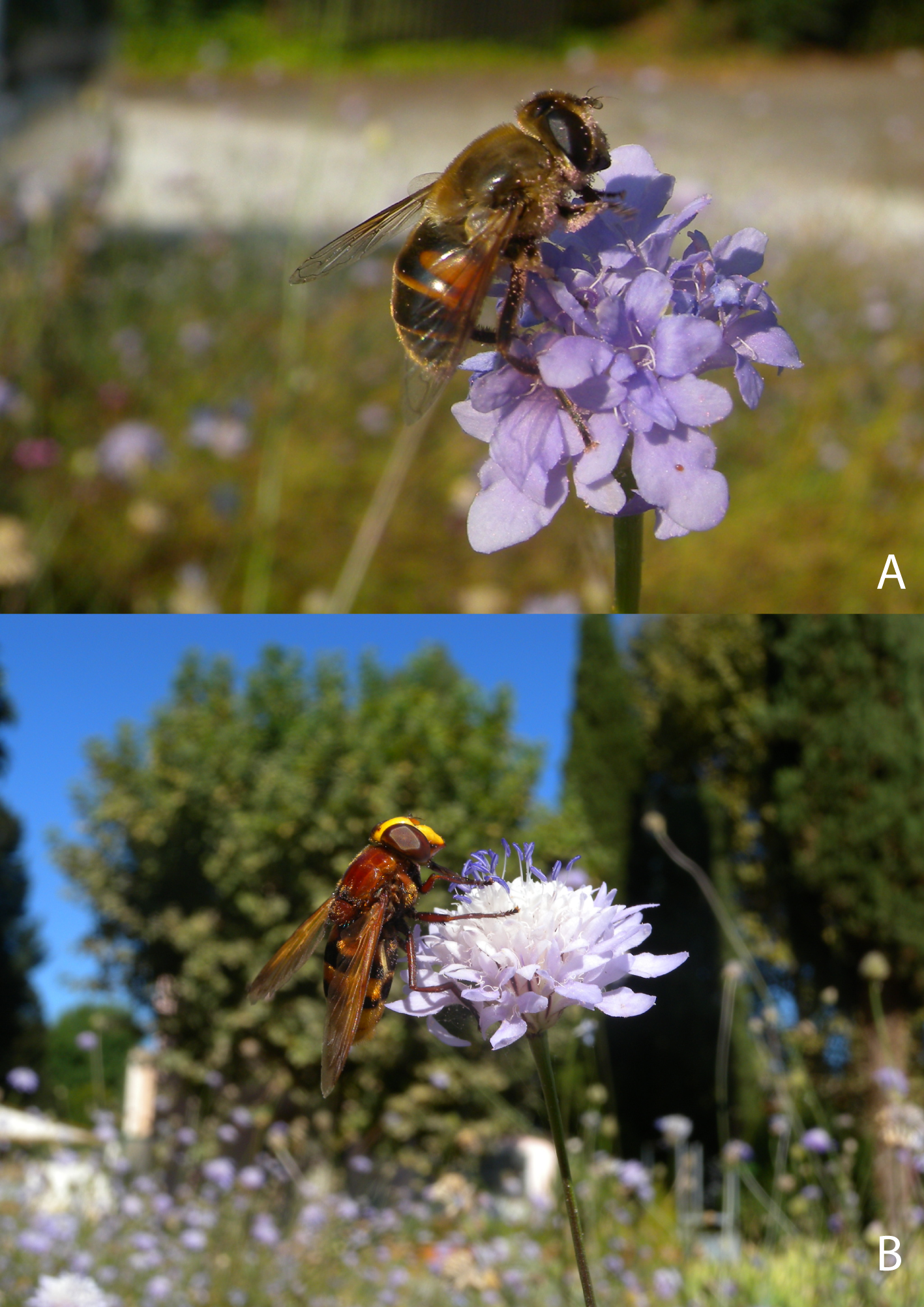

Supplement: Figure S4 — Cephalaria transsylvanica is an abundant source of pollen for different insect species, including hoverflies (Diptera, Syrphidae): (A) Eristalis tenax and (B) Volucella zonaria . (TIF) [file pone.0093153.s004.tif]
