# Supplementary material for: Cephalaria transsylvanica-Based Flower Strips as Potential Food Source for Bees during Dry Periods in European Mediterranean Basin Countries
Source: PLoS One. 2014 Mar 27;9(3):e93153. doi: 10.1371/journal.pone.0093153 (PMC3968061; doi:10.1371/journal.pone.0093153)
Supplement: Table S1 — Height of the Cephalaria transsylvanica inflorescences during the summer of the two cultivation years (2012–2013). (DOC) [file pone.0093153.s005.doc]

**Table S1**

| **Months** | **Height of the inflorescences from the ground (cm)**  **(mean ± SD)** | | | |
| --- | --- | --- | --- | --- |
|  | **2012** | | **2013** | |
|  | **Maximum** | **Average** | **Maximum** | **Average** |
| May | 107 ± 5 | 75 ± 4 | 94 ± 4 | 68 ± 5 |
| June | 124 ± 7 | 86 ± 5 | 112 ± 5 | 81± 5 |
| July | 135 ± 6 | 94 ± 5 | 127 ± 5 | 92 ± 7 |
| August | 143 ± 7 | 102 ± 7 | 138 ± 6 | 98 ± 7 |
| September | 137± 6 | 92 ± 5 | 126 ± 5 | 87 ± 5 |
